# Effects of virtual nature scenes and imagery dialogue intervention on academic anxiety in college students

**DOI:** 10.3389/fpsyg.2026.1826402

**Published:** 2026-08-21

**Authors:** Hao Fang, Ying Ran, Yifan Xu, Ran Cheng, Hongyun Guo, Qinling Dai, Xing Chen

**Affiliations:** 1 School of Art and Design, Wuhan Institute of Technology, Wuhan, China; 2 School of Arts and Communication, China University of Geosciences (Wuhan), Wuhan, China; 3 School of Marxism, China University of Geosciences (Wuhan), Wuhan, China; 4 School of Art and Design, Southwest Forestry University, Kunming, China; 5 Beijing Advanced Innovation Center for Biomedical Engineering, School of Engineering Medicine, Beihang University, Beijing, China

**Keywords:** academic anxiety, college students, imagery dialogue, virtual nature, virtual reality

## Abstract

**Background:**

Academic anxiety is increasingly prevalent among college students and can negatively affect both academic performance and mental well-being. Virtual reality-based nature exposure has emerged as a promising approach for emotional regulation. This study examined whether integrating imagery dialogue with virtual natural environments could enhance the effectiveness of anxiety interventions.

**Methods:**

A 2 (time: pre-test, post-test) × 4 (group: imagery dialogue-based virtual nature, virtual nature, imagery dialogue, control) mixed experimental design was adopted. Sixty college students with high academic anxiety were randomly assigned to one of the four groups and participated in a three-week intervention. Subjective measures, including the Academic Anxiety Questionnaire and the State Anxiety subscale of the State–Trait Anxiety Inventory, and physiological indicators, including heart rate and skin temperature, were collected before and after the intervention.

**Results:**

All intervention groups showed significant reductions in academic anxiety from baseline, while the control group showed no significant pre-post change. The combined group showed the largest descriptive reduction, but superiority over single interventions was not established. Physiological indicators showed favorable pre-post changes, with significant time × group interactions for both indicators.

**Conclusion:**

Imagery dialogue, virtual nature exposure, and their combination may all contribute to reducing academic anxiety among college students, with the combined approach showing potential as a digital intervention strategy.

## 1 Introduction

### 1.1 Academic anxiety in college students

In recent years, anxiety among college students has become increasingly prominent, with academic anxiety emerging as a primary form of this issue. A systematic review and meta-analysis revealed that approximately one-third of undergraduates experience elevated levels of non-specific anxiety, indicating that anxiety burden among college students is both widespread and persistent (Ahmed et al., 2023). Academic anxiety refers to a negatively valenced high-arousal emotion experienced in school contexts and is one of the most common negative emotions in academic settings (Zhang T. et al., 2025). Many college students experience academic anxiety to varying degrees, which not only reduces learning efficiency but also leads to negative psychological states such as depression and social withdrawal (Beiter et al., 2015).

At the theoretical level, the control-value theory suggests that anxiety is most likely triggered when students perceive low control over academic tasks but attribute high value to the outcomes. This in turn undermines motivation and self-regulation (Pekrun, 2024). Research on the relationship between academic emotions and learning outcomes has shown that positive emotions are generally associated with better academic performance, whereas anxiety correlates with reduced outcomes (Tan et al., 2021). Beyond psychological mechanisms, anxiety is also linked to imbalances in autonomic nervous system functioning. Multiple reviews have found that anxiety is associated with reduced heart rate variability, sympathetic dominance (e.g., reduced RMSSD/HF), and changes in stress-related EEG rhythms (Tomasi et al., 2024). Prolonged academic anxiety can activate the sympathetic nervous system and the hypothalamic-pituitary-adrenal axis, resulting in physiological responses such as increased heart rate, gastrointestinal discomfort, and sleep disturbances (Wang et al., 2023). Studies have also shown that acute stress rapidly activates the sympathetic-adrenal-medullary system, releasing adrenaline and noradrenaline, leading to an increased heart rate. If stress persists, the hypothalamic-pituitary-adrenal axis releases cortisol to sustain the stress response (Chu et al., 2024). Based on this evidence, academic anxiety not only seriously affects college students’ psychological well-being but also produces measurable physiological stress markers, underscoring the importance of finding effective intervention strategies.

### 1.2 Intervention effects of nature and virtual nature

According to attention restoration theory and stress recovery theory, exposure to natural environments can restore directed attention, reduce mental fatigue, and improve negative emotions (Liu et al., 2024; Ulrich et al., 1991). Multiple studies have demonstrated the significant positive effects of nature on individuals’ mental and physical health (Wendelboe-Nelson et al., 2019; Bowler et al., 2010). Bellini and colleagues proposed that restorative environments help reduce psychological stress, alleviate negative emotions, and facilitate psychological and physiological recovery (Bellini et al., 2023). Wang et al. (2020) found through comparisons of indoor and outdoor natural scenes that college students exhibited significantly improved physiological indicators in forested outdoor environments, with more pronounced changes in aquatic and woodland landscapes.

Ecotherapy emphasizes that contact with green environments can significantly improve mood and reduce anxiety. In recent years, virtual reality (VR) technology has been used to simulate natural environments to overcome the limitations of real-world exposure (Carl et al., 2019; Opriş et al., 2012). Previous studies have found that immersion and presence are the core mechanisms by which virtual reality exerts its psychological intervention effect (Slater, 2009; Witmer and Singer, 1998). This high presence can “trick” the brain’s perception system and trigger psychological recovery mechanisms similar to those in real natural environments (Li et al., 2023). Another randomized controlled trial found that several weeks of daily exposure to virtual nature significantly reduced anxiety arousal and worry among college students (Browning et al., 2023). More importantly, nature scenes replicated in VR environments have a positive effect on mental health and well-being among college students (Hubbard et al., 2025). Collectively, these findings support virtual nature interventions as an emerging psychological regulation tool capable of effectively alleviating anxiety and negative emotions in the short term, providing a scientific foundation for interventions targeting academic anxiety.

### 1.3 Imagery dialogue technique and nature imagery

Imagery communication psychotherapy is a psychological intervention that centers on visual symbolism and unconscious work. Rooted in Jungian analytic psychology, it incorporates elements from psychoanalysis, humanism, and Chinese philosophical traditions such as Buddhism and Daoism (Yang and Yuan, 2021). Studies have shown that guided imagery meditation or similar imagery-based therapies can significantly reduce physiological arousal indicators (e.g., heart rate) and negative emotions, suggesting comparable psycho-physiological benefits in alleviating anxiety (Jo et al., 2024). Further research indicates that multisensory art tasks can activate neural networks associated with emotional regulation (Han et al., 2024), consistent with the body–mind model proposed by Czamanski-Cohen and Weihs. This model posits that emotional homeostasis and mind–body integration can be achieved through sensory engagement and symbolic expression (Czamanski-Cohen and Weihs, 2016).

Integrating imagery dialogue into the design of virtual nature scenes offers both intervention potential and theoretical innovation. On the one hand, immersive VR environments provide a “symbolic space” where unconscious content can be projected and explored (Rossi et al., 2025). On the other hand, nature imagery such as mountains, water, and animals carries rich archetypal meaning. Users can assign personal symbolic value to these elements based on their individual experiences. Georgieva and Georgiev noted that VR environments help individuals engage in deeper self-reflection and reinterpret adversity, thereby reconstructing personal narratives with new meaning (Georgieva and Georgiev, 2020). When combined with imagery dialogue techniques, such narrative reconstruction helps college students reframe academic stress or anxiety experiences into positive self-stories, thereby achieving cognitive transformation and emotional regulation. Thus, incorporating imagery dialogue into virtual nature interventions not only enriches intervention approaches but also extends the theoretical boundaries of virtual reality psychotherapy, with considerable innovative and applied potential.

### 1.4 Imagery dialogue and virtual reality technology

Integrating imagery dialogue into the design of virtual nature scenes has both intervention potential and theoretical significance. More broadly, the integration of digital technologies with active emotion-regulation strategies has attracted increasing research attention as an approach to psychological intervention. For example, decoded EEG neurofeedback has been used to guide cognitive reappraisal training, indicating that technology-assisted systems may support the acquisition and implementation of cognitive emotion-regulation strategies (Li et al., 2024). Within this broader development, VR provides a particularly relevant platform for imagery-based interventions by supplying multisensory external cues and an immersive context. Recent research indicates that the multi-sensory immersion of VR can create stronger emotional resonance for guided imagery, significantly improving users’ relaxation responses and intervention experience by enhancing concentration (Bosman et al., 2024). When integrated with guided imagery, VR may compensate for or reduce the cognitive load required for individuals to construct internal images in traditional imagery therapy by providing high-fidelity external visual stimulation, thereby enhancing immersion and therapeutic efficacy in emotion regulation and stress management (Tarrant et al., 2018).

Although previous studies have preliminarily confirmed the therapeutic effect of VR-based guided imagery therapy on post-traumatic stress disorder (PTSD) symptoms (Rizzo and Shilling, 2017), this study proposes a completely new theoretical framework regarding the research population, technology combination mechanism, and application scenarios. Immersive virtual natural environments serve as a low-cognitive-load, high-safety “sensory container” consistent with stress recovery theory, while imagery dialogue provides a “cognitive script” for inner self-reflection and psychological narrative reconstruction. This novel protocol, which deeply binds environment-mediated passive relaxation with subconscious-driven active meaning reconstruction, offers a highly promising paradigm shift for short-term interventions for non-clinical anxiety. Validating the efficacy of this unique combined intervention model will expand the theoretical boundaries and practical application scenarios of virtual reality psychological intervention.

### 1.5 Hypotheses

Based on the above theoretical and empirical foundations, this study hypothesizes that virtual nature scenes based on imagery dialogue will effectively reduce academic anxiety among college students. Specifically, we propose that compared with stand-alone imagery dialogue or virtual nature interventions, the integrated approach will have a stronger intervention effect on academic anxiety.

## 2 Method

### 2.1 Participants

The participants were students from a university in Wuhan, China. A total of 200 Academic Anxiety Questionnaires (AAQ) were distributed, and 177 valid responses were collected after removing invalid ones, with an effective response rate of 88.5%. Given that the AAQ score is a continuous variable, this study used the in-sample relative grouping method (Clinton and Meester, 2019), a commonly used method in psychology, instead of the external clinical threshold, to effectively distinguish the anxiety group. Based on the distribution characteristics of the initial total score, individuals with scores higher than the “mean + 1 standard deviation” threshold (i.e., higher than 27.52 points) were defined as the high academic anxiety group, and 60 individuals (24 males, 36 females, aged 18–25 years) were randomly selected from this group as formal participants. They were then randomly assigned to three experimental groups and one control group (15 people in each group) according to the sex ratio. Baseline tests showed no significant differences among the four groups in sex, age, academic anxiety, skin temperature, or heart rate (*p* > 0.05), whereas baseline state-anxiety scores differed across groups, F (3, 56) = 4.20, *p* = 0.009.

To control for additional variables and ensure the smooth implementation of the VR intervention, this study established strict inclusion and exclusion criteria for participants:

1. Physiological characteristics: normal visual acuity or corrected visual acuity, no color blindness or color weakness, and all participants were right-handed;
2. Clinical history: no personal or family history of mental/neurological diseases;
3. Equipment tolerance: participants in the VR intervention group were required to be able to adapt to wearing the immersive head-mounted display and not experience severe VR motion sickness for at least 15 min during the intervention.

Before the formal start of the experiment, all participants were informed of the detailed experimental procedures and potential risks and voluntarily signed a written informed consent form.

### 2.2 Experimental design and procedure

#### 2.2.1 Experimental design

This study employed a 2 (time: pre-test, post-test) × 4 (groups: imagery dialogue group, virtual nature group, imagery dialogue-based virtual nature group, control group) mixed design. Time was a within-subjects factor, and group was a between-subjects factor. Dependent variables included state anxiety score, academic anxiety score, heart rate, and skin temperature.

#### 2.2.2 Procedure

A pretest-posttest design was employed. Assessments were conducted before the start of the intervention (baseline) and after the three-week intervention period. During the baseline assessment, participants completed standardized questionnaires to measure initial anxiety and related psychological indicators. After the intervention, the same questionnaires and physiological indicators were used to assess changes again. Specific measurement procedures: (1) Calm phase: before the intervention began, the participants needed to calm themselves down for 3 min. (2) Stress phase: after the participants calmed down, they needed to watch a video on academic anxiety for about 4 min on a computer screen to induce academic anxiety. The anxiety video was randomly assigned each time. (3) Measurement phase: after the academic anxiety was induced, the participants completed the Academic Anxiety Questionnaire and the State Anxiety subscale of the State–Trait Anxiety Inventory (STAI-S), and wore a wrist-worn physiological sensor to record skin temperature and heart rate within 3 min.

The intervention lasted for 3 weeks, led by a professional psychotherapist, twice a week for 15 min each time. The program integrated three main techniques, each grounded in a relevant theoretical framework: (1) Modeling aimed to promote adaptive emotion-al coping by observing and replicating positive behaviors, using demonstration to facilitate learning (Heyn et al., 2023). (2) Relaxation training sought to reduce physiological arousal through breath control, muscle relaxation, and other techniques grounded in the relaxation response (Luo et al., 2024). (3) Attention training focused on correcting attention bias and improving present-moment awareness via practices such as mindfulness meditation, thereby reducing hypervigilance to anxiety triggers and enhancing cognitive control (Fodor et al., 2020; Gupta et al., 2025).

The three-week protocol was organized around three components corresponding to emotional, somatic, and cognitive-attentional responses to academic anxiety. Week 1 used imagery-based modeling, in which the calm and stable qualities of a lake served as a symbolic reference for observing emotional fluctuations without immediate reaction. Week 2 focused on bodily awareness and breathing-based relaxation through mountain-path imagery. Week 3 used grassland imagery to support breath-focused attention and present-moment awareness. The weekly content and targeted psychological processes are summarized in Table 1.

**Table 1:** Three-week intervention content and corresponding psychological mechanisms.

Week	Intervention content	Targeted psychological process
Week 1	Imagery-based modeling using a lake scene: participants use the calm and stable qualities of the lake as a symbolic reference for observing emotional fluctuations without immediately reacting to them.	Symbolic modeling and emotional non-reactivity
Week 2	Body-focused relaxation using a mountain-path scene: participants attend to bodily discomfort and coordinate their attention with breathing during the imagined climbing process.	Somatic awareness and relaxation
Week 3	Attention training using a grassland scene: participants redirect attention to breathing and bodily sensations within an open natural setting.	Attentional refocusing and present-moment awareness

**Figure 1:**
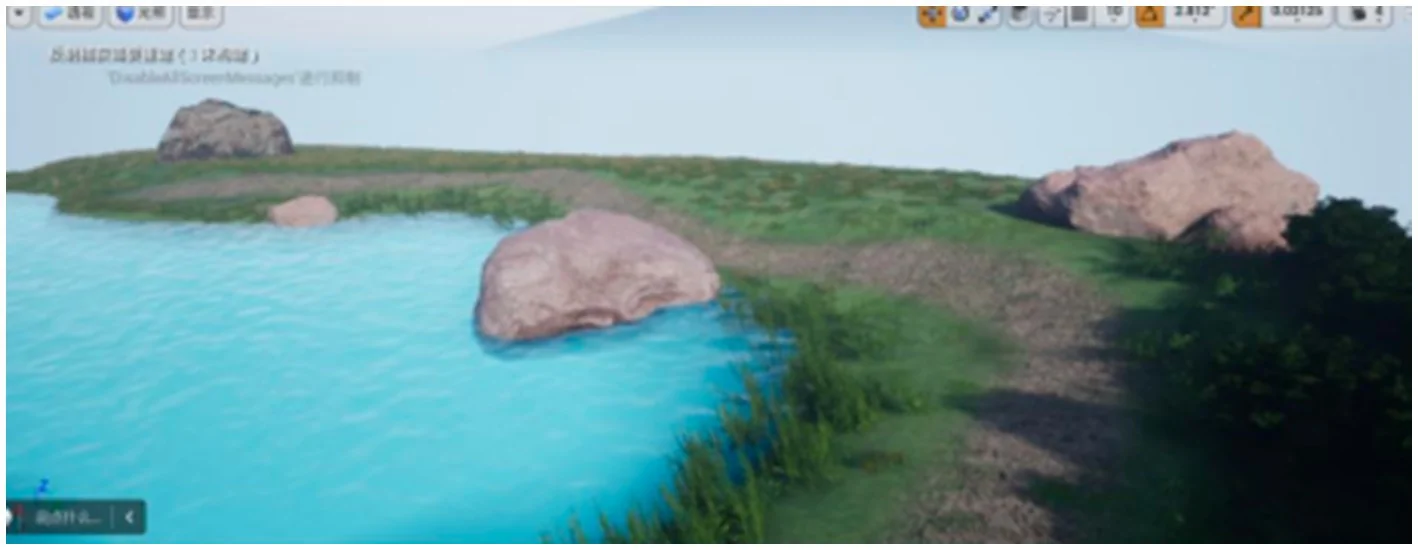
Virtual nature scene lake map.

**Figure 2:**
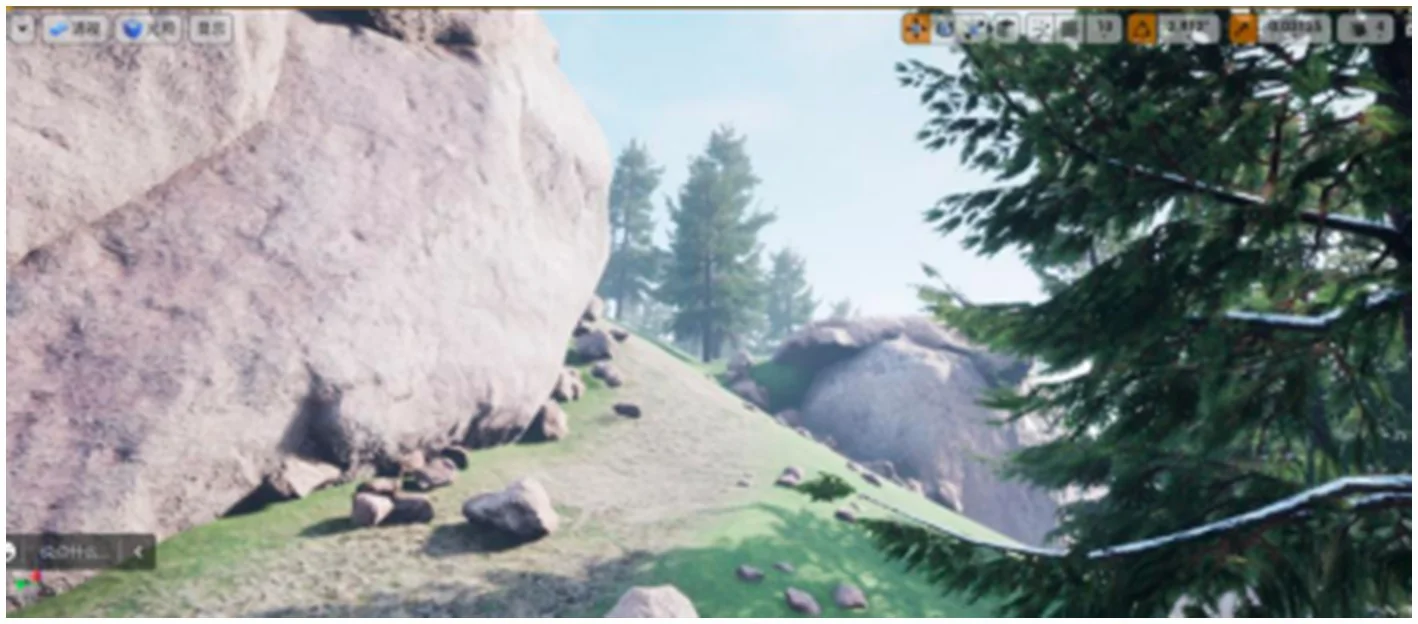
Virtual nature scene mountain road map.

**Figure 3:**
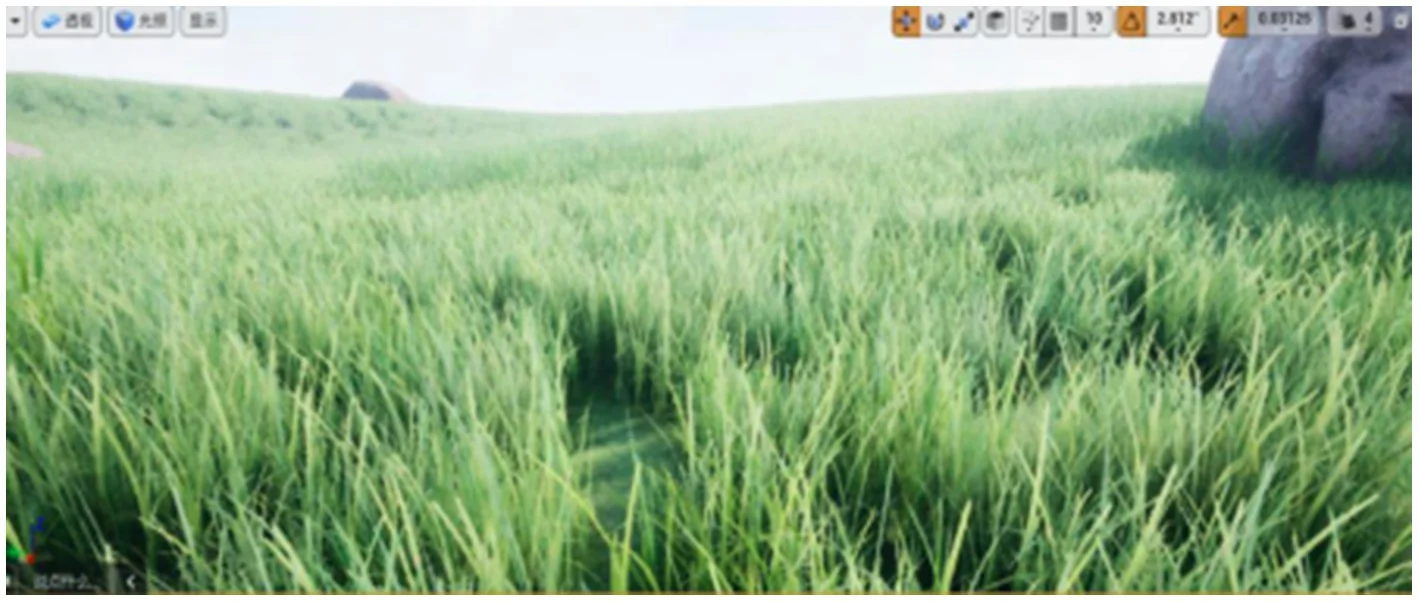
Virtual nature scene grassland map.

The specific time allocation and operation for each intervention group are as follows:

1. Imagery dialogue group (15 min)
2. Adaptation and preparation period (3 min): participants sit comfortably in a quiet laboratory chair and close their eyes. No interventional speech is used at this time. Participants adjust their state through deep breathing, adapting to the quiet environment and the natural white noise played by the system, gradually drawing their attention inward.
3. Guided intervention (10 min): the therapist leads the participants in imagery dialogue therapy without external visual stimulation. Under the guidance of the instructions, participants actively construct specific natural scenes in their minds (such as imagining a calm lake), and experience internal emotional projection based on the content of the speech (such as hearing “watching emotions flow through the body like lake water”), relying on pure internal imagery processing to achieve psychological suggestion.
4. Awareness and relaxation period (2 min): the prompting stops, and participants are asked to continue keeping their eyes closed, performing deep breathing and limb relaxation to calm their emotions amidst the natural white noise. Afterward, participants slowly open their eyes upon hearing the prompt, completing one intervention session.
5. Virtual nature group (15 min)
6. Adaptation and exploration period (3 min): participants wear VR headsets and enter a specific virtual natural scene (e.g., a lake). No voice intervention is provided. Participants attempt to adapt to the dynamic VR environment and natural sounds (e.g., the sound of flowing water or wind) by gently turning their heads to explore the 360-degree environment and making slight movements.
7. Unguided VR exposure (10 min): high-fidelity pure VR environment exposure is maintained, with no guided imagery played throughout. Participants freely observe and immerse themselves in the three-dimensional space without interference, focusing their gaze on natural elements in the environment (e.g., a dynamic viewpoint locked on the water surface). This stage relies entirely on the highly realistic immersive environment and visual exposure to natural elements to induce instinctive relaxation and attention shift in the participants.
8. Awareness and Relaxation Period (2 min): the scene elements remain unchanged. Participants are instructed to maintain deep breathing and physical relaxation within the VR scene to calm their emotions. The VR headset is then removed, completing the single intervention.
9. Virtual nature group based on imagery dialogue (15 min)
10. Adaptation and exploration period (3 min): participants wear VR headsets and enter a specific virtual natural scene (e.g., a lake) for the week. No voice intervention is provided. Participants attempt to adapt to the dynamic VR environment and ambient white noise by gently turning their heads to explore the 360-degree environment and making slight movements.
11. Synchronous guided intervention (10 min): the imagery dialogue voice-over script is activated in the background. The system simultaneously provides VR visual exposure and imagery voice guidance. Following the guidance, participants anchor their gaze on specific VR elements (e.g., upon hearing “watching emotions flow through the body like lake water,” focusing their visual attention on the dynamic water surface), achieving a two-way superposition of visual immersion and psychological suggestion.
12. Awareness and relaxation period (2 min): the voice-over stops, and participants are instructed to maintain deep breathing and physical relaxation within the VR scene to calm their emotions. The VR headset is then removed, completing one intervention session (see Table 2; Figures 1–3).

**Table 2:** Intervention environment.

Intervention phase	Types of responses	Imagery dialogue group	Virtual nature group	Imagery dialogue-based virtual nature group
Week 1	Emotional response	Imagery of a “Lake”: close your eyes and imagine a calm lake surface. Auditory guidance: “Let your emotions flow through your body like lake water.”	Lake scene walkthrough: freely observe the lake and shoreline elements while wearing a VR headset. No voice guidance.	VR lake + voice guidance Immerse yourself in the VR lake scene, with synchronized “Lake” instructions. Imagine the lake surface as your inner self, and the rocks at the bottom as your anxieties.
Week 2	Physical response	Imagery of a “Mountain Path”: close your eyes and imagine climbing a steep mountain path. Auditory guidance: “Relieve the physical discomfort of each step with your breath.”	Mountain road scene walkthrough: explore narrow, rugged, and steep mountain roads while wearing a VR headset. No voice guidance.	VR mountain path + voice guidance Immerse yourself in the VR mountain path scene, with synchronized “Mountain Path” instructions. Imagine the boulders before you as real obstacles, and alleviate the feeling of oppression through the action of “climbing.”
Week 3	Cognitive response	Imagery of a “Grassland”: close your eyes and imagine yourself on a vast grassland. Auditory guidance: “Focus on your breathing, and feel the separation and relaxation of body and mind with the gentle breeze.”	Grassland scene walkthrough: enjoy 360-degree observation of open grasslands while wearing a VR headset. No voice guidance.	VR grassland + voice guidance Immerse yourself in the VR grassland scene, with synchronized “Grassland” instructions. Take a deep breath in the boundless sea of grass, and use the immersive audiovisual experience to resolve your inner conflicts.

### 2.3 Research instruments

#### 2.3.1 Research equipment

This study utilized the Oculus Quest 2 headset for experiencing the VR scenarios. The Oculus Quest 2 uses an LCD display, equipped with a Qualcomm Snapdragon XR2 processor, and has 6 GB of RAM. It offers a display resolution of 1832 × 1920 per eye and 3664 × 1920 for both eyes, supporting refresh rates of 72 Hz and 90 Hz. It tracks user movement without requiring external sensors, and its tracking is relatively accurate. The 3D models for the experimental materials were created using Autodesk 3ds Max, with texture mapping performed in Photoshop, and the development executed in the Unreal Engine 4 game engine. Additionally, the E4 wristband biosensor was used to collect physiological indicators, capable of recording skin temperature, electrodermal activity, and heart rate data.

#### 2.3.2 Research materials

Previous studies indicate that emotional induction is more effective when both visual and auditory stimuli are used in dynamic presentations, generating stronger subjective experiences and physiological changes (Oh et al., 2025). This study searched multimedia platforms across the internet and selected 10 videos highly relevant to academic stress in higher education. The videos covered realistic final exam countdown scenarios, first-person perspectives of intensive study tasks, and competitive pressure situations, with each video approximately 4 min long. Twenty students with high levels of academic anxiety, exhibiting similar characteristics to the experimental participants, were selected to rate the video clips. The hit rate was used to evaluate emotional specificity—i.e., the proportion of raters who scored the target emotion (anxiety) at least one point higher than all other emotions. A higher hit rate indicates better specificity (Zhang Y et al., 2021). Ratings used a 9-point Likert scale ranging from 0 (not at all) to 8 (extremely intense). The video clips with the highest anxiety scores and hit rates were selected as experimental materials. All clips were processed to ensure consistent duration, screen ratio, and visual effects. Validation results showed significant anxiety arousal effects (*p* < 0.05) with no significant differences between videos (*p* > 0.05). Therefore, the anxiety-inducing videos were deemed suitable for use in the stress-induction phase of the formal experiment.

To ensure the intervention materials possess high ecological validity and intervention specificity, the imagery dialogue, virtual natural environment, and imagery-virtual synesthesia paradigm used in this study underwent a dual verification process, from theoretical derivation to expert review. The expert team included academic leaders and senior consultants from a psychological research institute in Wuhan. The material development process followed these steps: (1) identify emotional intentions and symbolic natural scenes; (2) write imagery dialogue scripts tailored to academic anxiety; (3) develop virtual nature scenes based on these scripts; (4) determine the duration of the intervention cycle.

#### 2.3.3 Measures

1. Academic Anxiety Questionnaire

Academic anxiety was assessed using the seven-item academic-anxiety subscale of the Academic Emotions Questionnaire developed by Dong and Yu (2007), which is referred to in the present study as the Academic Anxiety Questionnaire. Items were rated from 1 (strongly disagree) to 5 (strongly agree), yielding a total score ranging from 7 to 35, with higher scores indicating greater academic anxiety. The original questionnaire demonstrated satisfactory reliability and validity. In the present study, Cronbach’s α was 0.81.

1. State Anxiety Subscale of the State–Trait Anxiety Inventory

State anxiety was assessed using the 20-item State Anxiety subscale of the State–Trait Anxiety Inventory (STAI), originally developed by Spielberger et al. (1970). The Form Y version (Spielberger et al., 1983), specifically the Chinese version revised for Chinese college students by Li and Qian (1995), was used in the present study. Items were rated on a 4-point Likert scale, yielding total scores ranging from 20 to 80, with higher scores indicating greater state anxiety. In the present study, Cronbach’s α was 0.861.

1. Physiological emotion indicators

- Heart rate: regulated by the autonomic nervous system, is a direct reflection of physiological arousal (Wascher, 2021). It is one of the most widely used physiological indicators in emotion monitoring (Lal et al., 2024). Empirical studies show that elevated heart rate relative to baseline is associated with high-arousal emotions such as fear, anxiety, and excitement, while parasympathetic dominance in relaxed states reduces heart rate (Raz and Lahad, 2022).
- Skin temperature: modulated by peripheral blood flow via autonomic regulation, serves as a potential physiological indicator of emotional arousal (Shelepenkov et al., 2025). During stress or fear, sympathetic activation causes vasoconstriction, leading to lower skin temperatures, especially in extremities like the hands. Conversely, in pleasant and relaxed states, peripheral circulation improves and skin temperature rises (Iwase et al., 2017). Recent thermal imaging research shows that basic emotions induce temperature changes in specific body regions—for example, chest skin temperature rises significantly during fear, surprise, and joy, and these changes correlate closely with heart rate (Shelepenkov et al., 2025).

#### 2.3.4 Data analysis

All data analyses were conducted using SPSS statistical software. Descriptive statistics were used to summarize the baseline characteristics of participants and baseline equivalence among groups was examined using appropriate statistical tests. To explore the impact of different intervention modalities, this study conducted mixed ANOVAs of 2 (measurement time) × 4 (experimental group) based on differences in dependent variables (academic anxiety score, state anxiety score, skin temperature, and heart rate). For indicators showing significant time × group interactions, simple-effects tests were conducted with Bonferroni correction. For indicators with non-significant interactions but significant group main effects, post-hoc multiple comparisons were performed using Tukey’s HSD test. The statistical significance threshold for all tests was *p* < 0.05.

## 3 Results

### 3.1 Descriptive statistics of scores for each group in the pre-test and post-test

**Figure 4:**
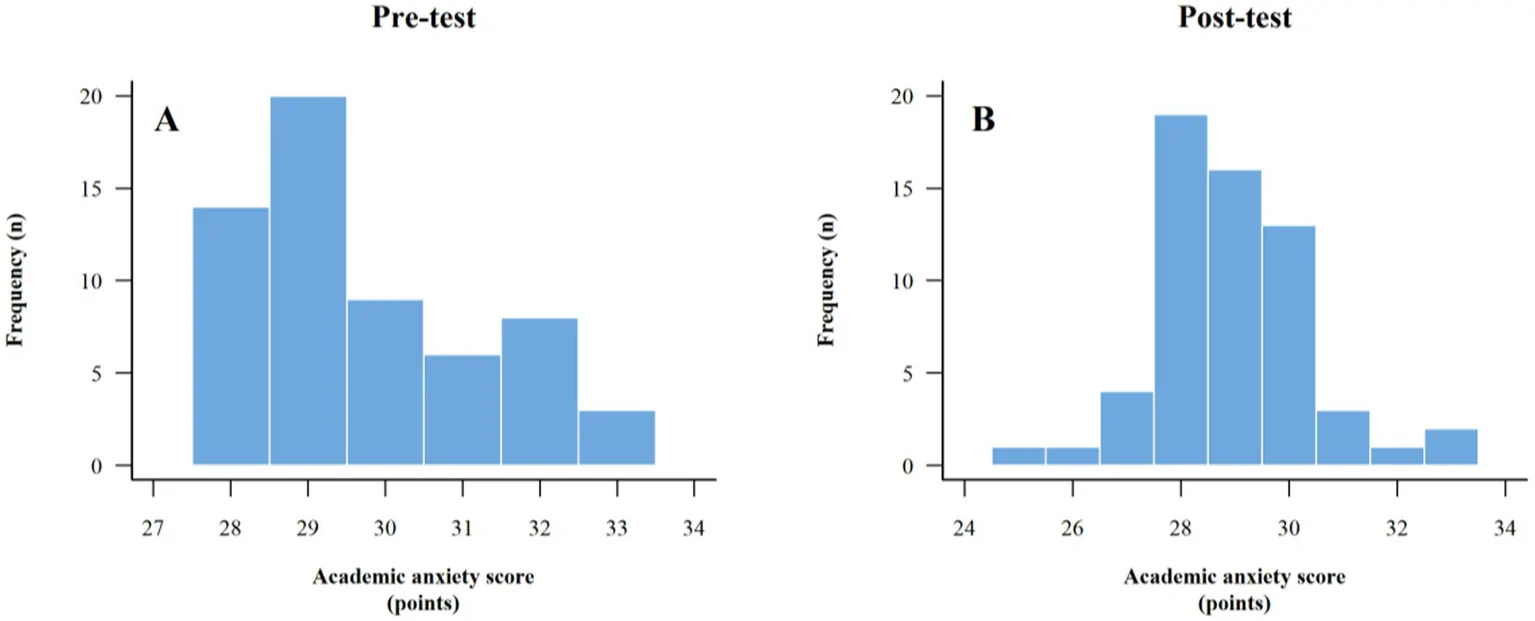
Distribution of academic anxiety questionnaire scores at pre-test and post-test in the total sample. **(A)** Pooled pre-test scores across all four groups; **(B)** pooled post-test scores across all four groups.

This study conducted descriptive statistics on the pre- and post-test performance of the four groups (imagery dialogue group, virtual nature group, imagery dialogue-based virtual nature group, and no-intervention control group) on four outcome indicators: state anxiety, academic anxiety, skin temperature, and heart rate (see Table 3). At the descriptive level, the three intervention groups generally showed reductions in state anxiety, academic anxiety, and heart rate after the intervention, together with increases in skin temperature, whereas the control group showed relatively small pre-post changes. To complement these group-level descriptive statistics, Figure 4 presents the pooled frequency distributions of academic anxiety questionnaire scores for the total sample at pre-test and post-test. Group differences and time × group effects were further examined in the subsequent mixed ANOVA analyses.

**Table 3:** Descriptive statistics of scores for each group in the pre-test and post-test.

Groups	Sample size (*N*)	State anxiety score		Academic anxiety score		Skin temperature (°C)		Heart rate (beats/min)	
		Pre-test (*M* ± *SD*)	Post-test (*M* ± *SD*)	Pre-test (*M* ± *SD*)	Post-test (*M* ± *SD*)	Pre-test (*M* ± *SD*)	Post-test (*M* ± *SD*)	Pre-test (*M* ± *SD*)	Post-test (*M* ± *SD*)
Imagery dialogue group	15	44.73 ± 7.07	43.07 ± 7.59	29.93 ± 1.58	29.07 ± 1.03	24.91 ± 1.88	25.28 ± 1.51	97.42 ± 6.96	92.14 ± 7.21
Virtual nature group	15	53.00 ± 11.21	51.93 ± 10.95	29.53 ± 1.40	28.53 ± 1.18	24.69 ± 2.04	25.30 ± 1.63	94.74 ± 10.29	91.06 ± 9.22
Imagery dialogue-based virtual nature group	15	53.93 ± 12.02	52.27 ± 12.62	29.73 ± 1.58	28.20 ± 1.32	24.84 ± 1.90	26.00 ± 2.17	95.16 ± 6.80	88.98 ± 6.57
Control group (no intervention)	15	57.87 ± 10.70	57.60 ± 10.73	29.67 ± 1.63	29.93 ± 1.71	25.59 ± 2.30	25.57 ± 2.28	93.97 ± 7.98	95.40 ± 8.14
Overall	60	52.38 ± 11.24	51.22 ± 11.61	29.72 ± 1.51	28.93 ± 1.46	25.01 ± 2.02	25.54 ± 1.90	95.32 ± 8.03	91.89 ± 8.00

### 3.2 Analysis of anxiety scores in different groups before and after intervention

#### 3.2.1 State anxiety

Regarding state anxiety, the main effect of time was significant, *F* (1, 56) = 17.69, *p* < 0.001, partial *η*^2^ = 0.24, indicating that state-anxiety scores were lower at post-test than at pre-test when averaged across groups. The main effect of group was also significant, *F* (3, 56) = 4.54, *p* = 0.006, partial *η*^2^ = 0.19, indicating an overall difference in state-anxiety scores among the four groups. However, the time × group interaction was not significant, *F* (3, 56) = 1.43, *p* = 0.244, partial *η*^2^ = 0.07, indicating that the magnitude of pre-post change did not differ significantly across groups (see Table 4).

**Table 4:** Mixed ANOVA by group and time.

Source	STAI-S	AAQ
Time	*F*(1, 56) = 17.69 *p* < 0.001 partial *η*^2^ = 0.24	*F*(1, 56) = 36.55 *p* < 0.001 partial *η*^2^ = 0.39
Group	*F*(3, 56) = 4.54 *p* = 0.006 partial *η*^2^ = 0.19	*F*(3, 56) = 1.27 *p* = 0.293 partial *η*^2^ = 0.06
Time × Group	*F*(3, 56) = 1.43 *p* = 0.244 partial *η*^2^ = 0.07	*F*(3, 56) = 8.53 *p* < 0.001 partial *η*^2^ = 0.31

**Figure 5:**
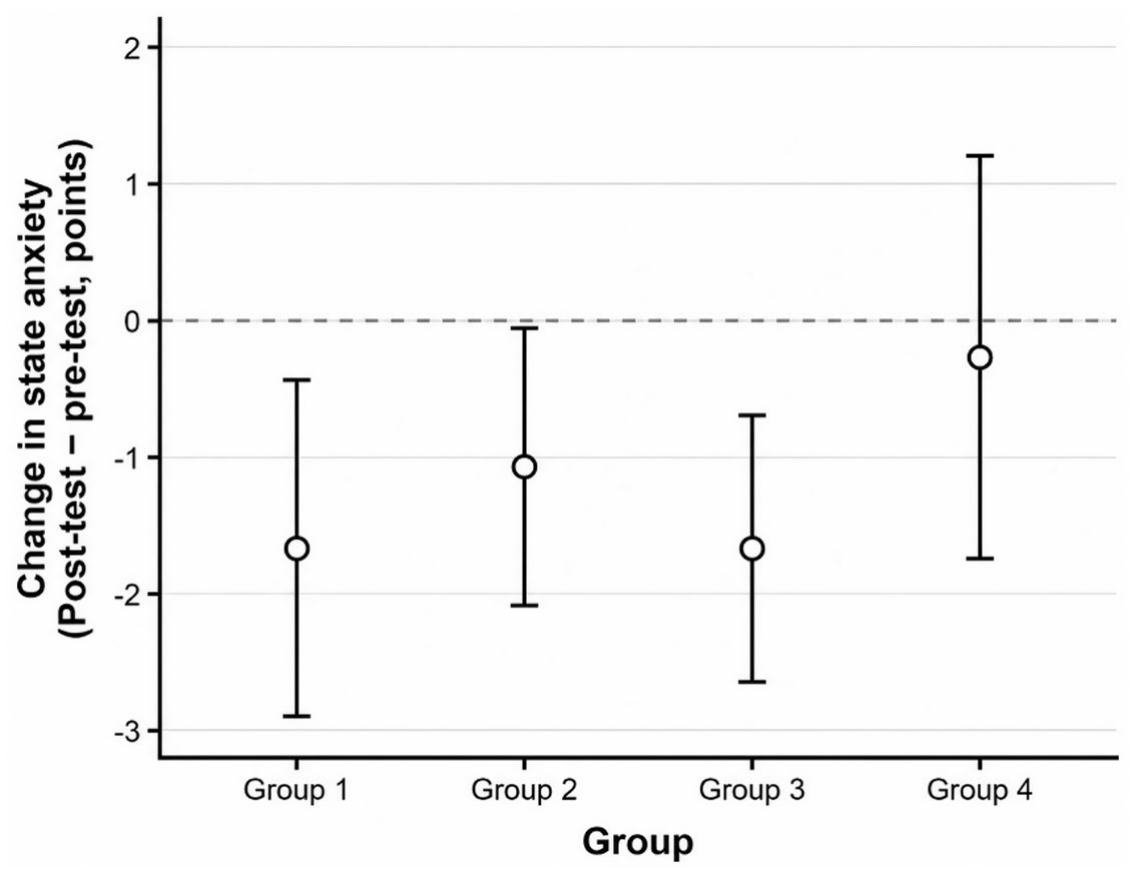
Descriptive changes in state anxiety from pre-test to post-test across groups. Values represent mean change scores calculated as post-test minus pre-test.

Given the significant main effect of group, Tukey’s HSD post-hoc comparisons were conducted to examine pairwise differences in state-anxiety scores averaged across the two measurement occasions. The results showed that the overall state-anxiety score of the imagery dialogue group was significantly lower than that of the control group (*MD* = −13.83, *p* = 0.003). However, the imagery dialogue group did not differ significantly from either the imagery dialogue-based virtual nature group (*MD* = −9.20, *p* = 0.088) or the virtual nature group (*MD* = −8.57, *p* = 0.125). No significant differences were observed between the virtual nature group and the control group (*MD* = −5.27, *p* = 0.519), between the imagery dialogue-based virtual nature group and the control group (*MD* = −4.63, *p* = 0.622), or between the virtual nature group and the imagery dialogue-based virtual nature group (*MD* = −0.63, *p* = 0.998; Table 5). Because the time × group interaction was not significant, these comparisons reflect overall group differences averaged across measurement occasions rather than differences in intervention-related change. Descriptively, the mean pre-post changes in state anxiety were −1.67, −1.07, −1.67, and −0.27 points for the imagery dialogue group, virtual nature group, imagery dialogue-based virtual nature group, and control group, respectively (Figure 5). These values are presented to visualize the descriptive pre-post patterns because the time × group interaction for state anxiety was not significant.

**Table 5:** *Post-hoc* multiple comparisons of state anxiety scale scores for each group.

(I) Conditions	(J) Conditions	Mean difference (I-J)	Standard error	*p*	95% confidence interval	
					Lower limit	Upper limit
Imagery dialogue group	Virtual nature group	−8.57	3.825	0.125	−18.70	1.56
	Imagery dialogue-based virtual nature group	−9.20	3.825	0.088	−19.33	0.93
	Control group (no intervention)	−13.83*	3.825	0.003	−23.96	−3.70
Virtual nature group	Imagery dialogue group	8.57	3.825	0.125	−1.56	18.70
	Imagery dialogue-based virtual nature group	−0.63	3.825	0.998	−10.76	9.50
	Control group (no intervention)	−5.27	3.825	0.519	−15.40	4.86
Imagery dialogue-based virtual nature group	Imagery dialogue group	9.20	3.825	0.088	−0.93	19.33
	Virtual nature group	0.63	3.825	0.998	−9.50	10.76
	Control group (no intervention)	−4.63	3.825	0.622	−14.76	5.50
Control group (no intervention)	Imagery dialogue group	13.83*	3.825	0.003	3.70	23.96
	Virtual nature group	5.27	3.825	0.519	−4.86	15.40
	Imagery dialogue-based virtual nature group	4.63	3.825	0.622	−5.50	14.76

#### 3.2.2 Academic anxiety

As shown in Table 4, the main effect of time on academic anxiety was significant, *F* (1, 56) = 36.55, *p* < 0.001, partial η^2^ = 0.39, indicating that the post-test score was significantly lower than the pre-test score. The main effect of group was not significant, *F* (3, 56) = 1.27, *p* = 0.293, partial η^2^ = 0.06, indicating that there was no significant difference in scores between the four groups in the pre-test and post-test. The interaction between group and time was significant, *F* (3, 56) = 8.53, *p* < 0.001, partial η^2^ = 0.31. This indicates that the main effect of time on academic anxiety varies significantly among different groups, therefore, a simple effects test is needed.

**Figure 6:**
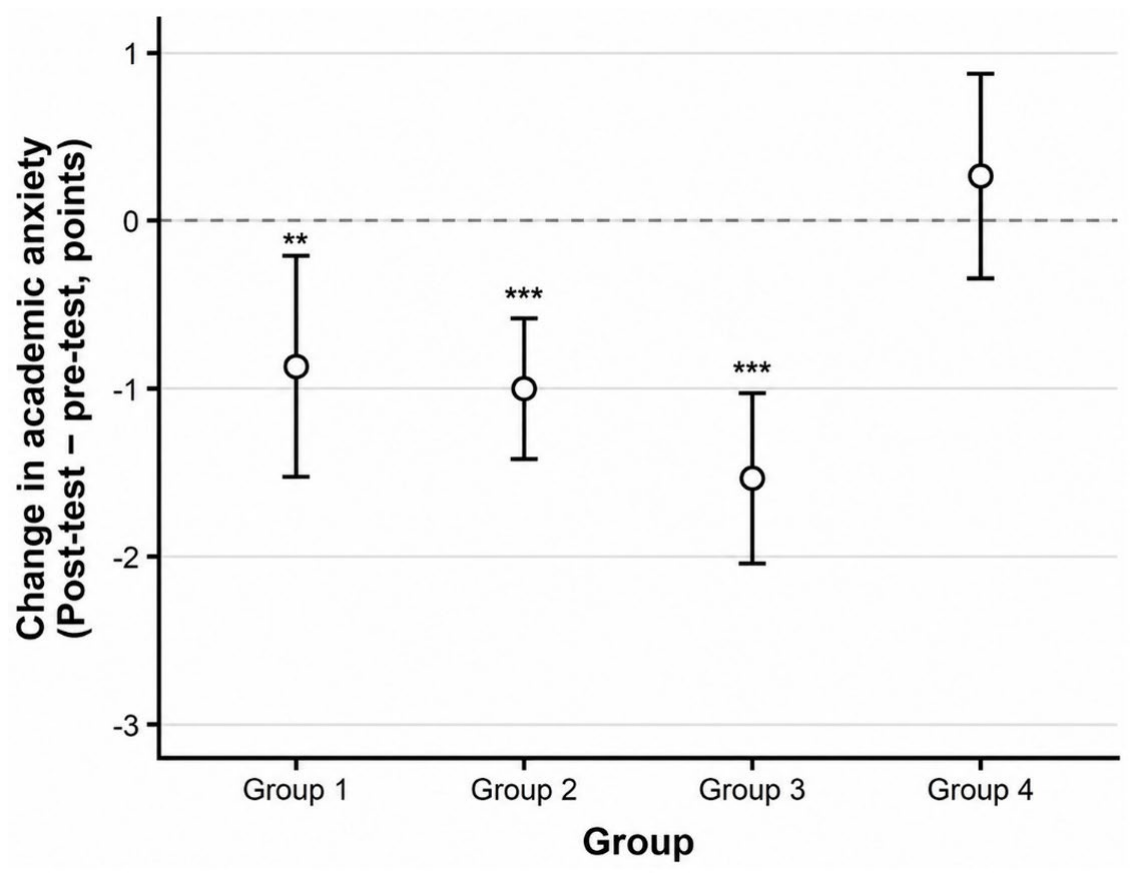
Changes in academic anxiety from pre-test to post-test across groups. Values represent mean change scores calculated as post-test minus pre-test. Asterisks denote Bonferroni-corrected within-group pre-post significance. *p < 0.05, **p < 0.01, ***p < 0.001.

Because the interaction effect between time and group was significant in academic anxiety scores, this study conducted a simple effects test (with Bonferroni correction) to further explore the specific differences between groups at different time points, fixing the variables between groups and analyzing differences over time. The results showed that academic anxiety decreased significantly in the imagery dialogue group (MD = −0.867, *p* = 0.001), the virtual nature group (MD = −1.000, *p* < 0.001), and the imagery dialogue-based virtual nature group (MD = −1.533, *p* < 0.001), whereas the control group showed no significant change (MD = 0.267, *p* = 0.308) (see Table 6). Thus, all three intervention groups showed significant pre-post reductions in academic anxiety, whereas the control group showed no significant change (Figure 6).

**Table 6:** Results of simple-effects tests on the academic anxiety scale for each group.

Groups	Comparison time	Mean difference	Standard error	*p*	95% confidence interval	
					Lower limit	Upper limit
Imagery dialogue group	Post-test − Pre-test	−0.867	0.259	=0.001	−1.386	−0.348
Virtual nature group	Post-test − Pre-test	−1.000	0.259	<0.001	−1.519	−0.481
Imagery dialogue-based virtual nature group	Post-test − Pre-test	−1.533	0.259	<0.001	−2.052	−1.014
Control group (no intervention)	Post-test − Pre-test	0.267	0.259	=0.308	−0.252	0.786

### 3.3 Analysis of physiological indicators in different groups before and after the intervention

Regarding heart rate, the main effect of time was significant (*F* (1, 56) = 23.70, *p* < 0.001, partial *η*^2^ = 0.30), indicating that the post-test heart rate was significantly lower than the pre-test heart rate. The main effect of group was not significant (*F* (3, 56) = 0.48, *p* = 0.699, partial *η*^2^ = 0.03), showing no significant overall difference between the four groups across measurement occasions. The interaction between group and time was significant (*F* (3, 56) = 5.82, *p* = 0.002, partial *η*^2^ = 0.24), indicating that the pre-post change in heart rate differed significantly across groups, thus requiring further simple effects testing (see Table 7). Regarding skin temperature, the main effect of time was significant, *F* (1, 56) = 18.94, *p* < 0.001, partial *η*^2^ = 0.25, indicating that the post-test skin temperature was significantly higher than pre-test skin temperature. The main effect of group was not significant, *F* (3, 56) = 0.31, *p* = 0.822, partial *η*^2^ = 0.02, indicating no significant overall group difference across measurement occasions. The interaction between group and time was significant, *F* (3, 56) = 4.13, *p* = 0.010, partial *η*^2^ = 0.18, indicating that the pre-post change in skin temperature differed significantly across groups, therefore, a simple effects test is needed (see Table 7).

**Table 7:** Mixed ANOVA by group and time.

Source	Heart rate (beats/min)	Skin temperature (°C)
Time	*F*(1, 56) = 23.70 *p* < 0.001 partial *η*^2^ = 0.30	*F*(1, 56) = 18.94 *p* < 0.001 partial *η*^2^ = 0.25
Group	*F*(3, 56) = 0.48 *p* = 0.699 partial *η*^2^ = 0.03	*F*(3, 56) = 0.31 *p* = 0.822 partial *η*^2^ = 0.02
Time × Group	*F*(3, 56) = 5.82 *p* = 0.002 partial *η*^2^ = 0.24	*F*(3, 56) = 4.13 *p* = 0.010 partial *η*^2^ = 0.18

**Figure 7:**
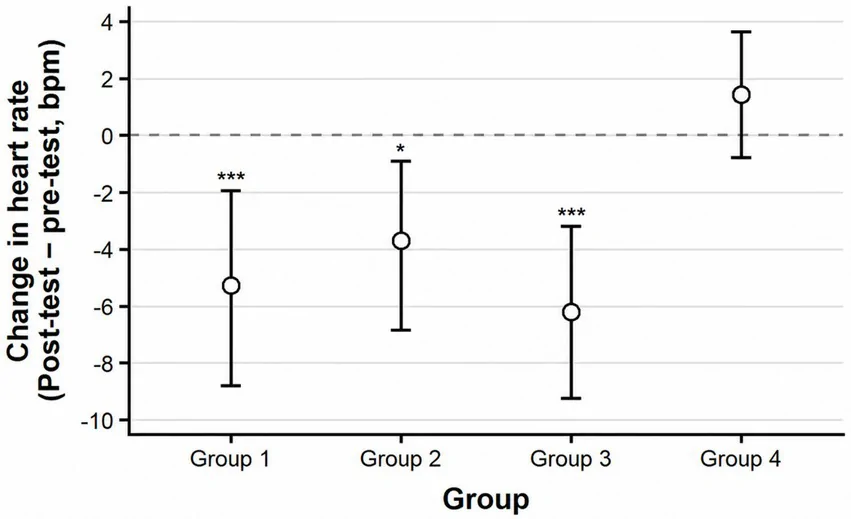
Changes in heart rate from pre-test to post-test across groups. Values represent mean change scores calculated as post-test minus pre-test. Asterisks denote Bonferroni-corrected within-group pre-post significance. *p < 0.05, **p < 0.01, ***p < 0.001.

Because heart rate showed a significant interaction effect between time and group, this study conducted a simple effects test (with Bonferroni correction) to further explore the specific differences between groups at different time points, fixing the variables between groups and analyzing differences over time. After the intervention, heart rate decreased significantly in the imagery dialogue group (MD = −5.277 beats/min, *p* < 0.001), the virtual nature group (MD = −3.683 beats/min, *p* = 0.011), and the imagery dialogue-based virtual nature group (MD = −6.175 beats/min, *p* < 0.001), whereas the control group showed no significant change (MD = 1.428 beats/min, *p* = 0.315; Table 8; Figure 7).

**Table 8:** Results of simple effect tests on heart rate for each group.

Factors	Comparison time	Mean difference	Standard error	*p*	95% confidence interval	
					Lower limit	Upper limit
Imagery dialogue group	Post-test - Pre-test	−5.277	1.408	< 0.001	−8.098	−2.457
Virtual nature group	Post-test - Pre-test	−3.683	1.408	= 0.011	−6.504	−0.863
Imagery dialogue-based virtual nature group	Post-test - Pre-test	−6.175	1.408	< 0.001	−8.996	−3.355
Control group (no intervention)	Post-test - Pre-test	1.428	1.408	= 0.315	−1.393	4.249

**Figure 8:**
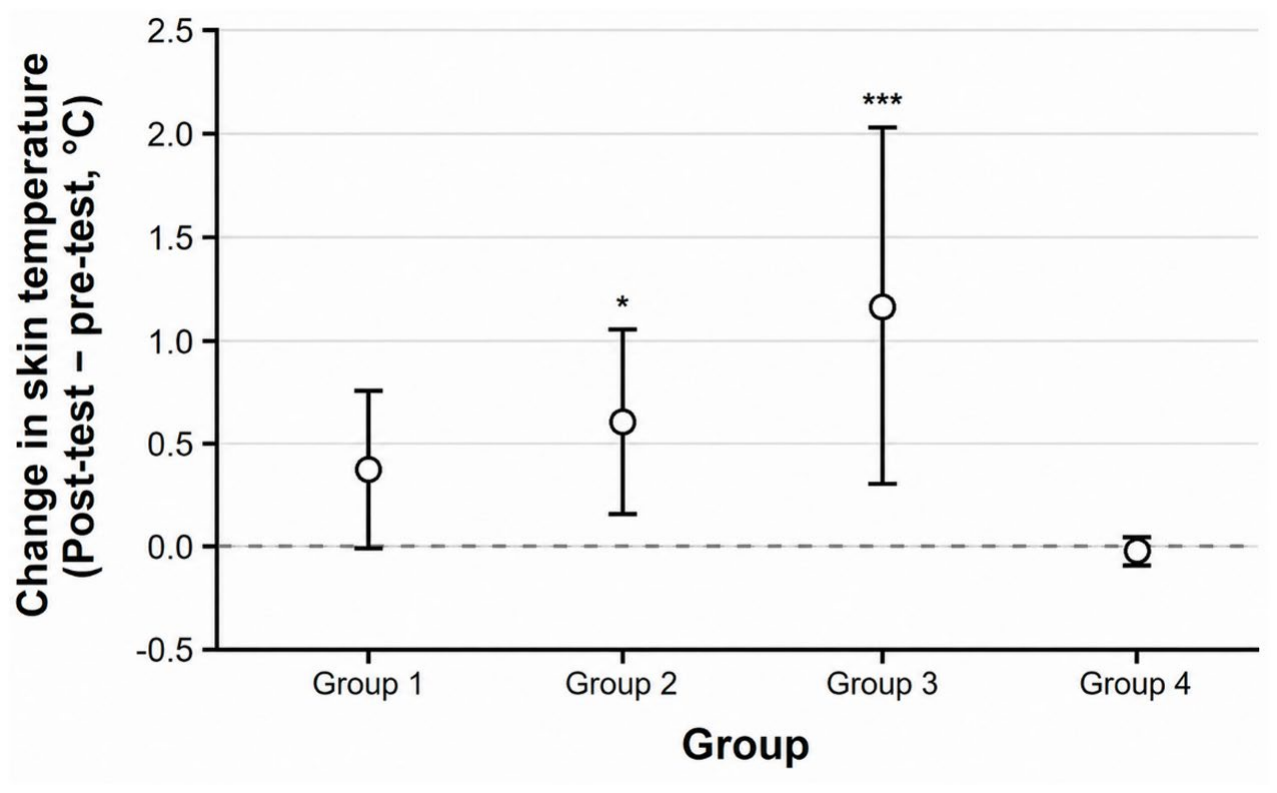
Changes in skin temperature from pre-test to post-test across groups. Values represent mean change scores calculated as post-test minus pre-test. Asterisks denote Bonferroni-corrected within-group pre-post significance. *p < 0.05, **p < 0.01, ***p < 0.001.

Similarly, skin temperature increased significantly in the virtual nature group (MD = 0.606°C, *p* = 0.016) and the imagery dialogue-based virtual nature group (MD = 1.161°C, *p* < 0.001), whereas the imagery dialogue group (MD = 0.374°C, *p* = 0.130) and the control group (MD = −0.023°C, *p* = 0.926) showed no significant change (Table 9; Figure 8).

**Table 9:** Results of simple-effects tests on wrist skin temperature for each group.

Groups	Comparison time	Mean difference	Standard error	*p*	95% Confidence interval	
					Lower limit	Upper limit
Imagery dialogue group	Post-test − Pre-test	0.374	0.243	= 0.130	−0.113	0.861
Virtual nature group	Post-test − Pre-test	0.606	0.243	= 0.016	0.119	1.093
Imagery dialogue-based virtual nature group	Post-test − Pre-test	1.161	0.243	< 0.001	0.673	1.648
Control group (no intervention)	Post-test − Pre-test	−0.023	0.243	= 0.926	−0.510	0.465

## 4 Discussion

### 4.1 Subjective anxiety intervention effects

This study used two measures of subjective anxiety: the Academic Anxiety Questionnaire and the State Anxiety Inventory. The former assesses students’ sustained anxiety experiences in academic contexts, focusing on concerns related to learning tasks and exam performance (Cassady et al., 2019), while the latter evaluates transient anxiety states in specific moments, reflecting immediate emotional responses (Valente et al., 2025). Combining these two tools allowed for a comprehensive evaluation of both situational and general emotional changes following the intervention.

Regarding academic anxiety, results showed that subjective academic anxiety levels significantly decreased across all intervention groups (the imagery dialogue group, the virtual nature group, and the imagery dialogue-based virtual nature group), whereas the control group did not exhibit notable changes. Of particular note is that the imagery dialogue-based virtual nature group showed the largest descriptive reduction in academic anxiety. This pattern suggests that integrating imagery dialogue with virtual nature scenes may have stronger anxiety-reducing potential than either method alone. However, because the reported simple-effects tests primarily examined within-group pre-post changes, the superiority of the combined intervention over the single interventions should be interpreted cautiously. Consistent with expectations, both imagery dialogue and virtual nature interventions alone were also effective compared to the control group, indicating that each approach has independent benefits in reducing anxiety.

The significant time × group interaction for academic anxiety indicates that the pre-post change in academic anxiety questionnaire scores differed across the four groups. The simple-effects tests showed that academic anxiety significantly decreased in the imagery dialogue group, the virtual nature group, and the imagery dialogue-based virtual nature group, whereas no significant pre-post change was observed in the control group. These findings suggest that all three intervention approaches were associated with reductions in academic anxiety, with the combined intervention showing the largest descriptive decrease. This pattern is consistent with the potential stress-reducing effects of immersive virtual nature. Such environments may alleviate academic stress by providing a restorative context and facilitating attentional disengagement from academic stressors (Chen et al., 2025). Guided imagery may operate primarily through cognitive-emotional mechanisms and has also been shown to reduce exam-related anxiety and stress responses (Zemla et al., 2023).

Regarding state anxiety, the time × group interaction was not significant, indicating that the pre-post change in state anxiety did not differ significantly across the four groups. Although the main effects of time and group were significant, these effects should be interpreted cautiously because the significant group effect reflected overall differences averaged across measurement occasions, rather than differential intervention-related changes. Therefore, the present results do not support a group-specific intervention effect on state anxiety. One possible explanation is that the state anxiety inventory captures relatively transient and broad emotional states that may be influenced by multiple situational factors beyond academic pressure. In contrast, academic anxiety appeared to be more sensitive to the present intervention protocol, which was specifically designed around academic stress.

### 4.2 Changes in physiological indicators

The study also examined whether the three intervention methods were associated with reductions in physiological arousal. The results partially supported this hypothesis. Heart rate significantly decreased in all three intervention groups, whereas no significant change was observed in the control group. For skin temperature, significant increases were observed in the virtual nature group and the imagery dialogue-based virtual nature group, while the imagery dialogue group showed only a non-significant increasing trend. Previous research indicates that parasympathetic activation leads to vasodilation and increased skin temperature, while sympathetic activation results in vasoconstriction and reduced temperature (Shelepenkov et al., 2025). A drop in heart rate typically signals reduced physiological stress (Suseno and Hastjarjo, 2023). Thus, the reductions in heart rate across the three intervention groups, together with the increases in skin temperature in the VR-related groups, suggest a relaxation response characterized by reduced physiological arousal. These findings align with physiological research on stress and relaxation, suggesting that immersion in virtual nature can induce a physical relaxation response, as evidenced by improved heart rate variability and reduced blood pressure (Vidyarini et al., 2024).

The lack of significant change in skin temperature in the imagery dialogue group may be due to the nature and intensity of the intervention. Imagery dialogue relies on participants’ internal imagination and symbolic interaction, lacking external sensory stimulation. As a result, it offers lower levels of immersion and realism. Research shows that multisensory immersion and a sense of “being there” are key to eliciting strong relaxation responses (Cieślik, 2025). For example, Pardini et al. (2024) found that immersive VR environments were more effective than pure imagination conditions in reducing subjective anxiety and tension during progressive muscle relaxation in college students. This implies that purely internal imagery-based interventions (like imagery dialogue) may insufficiently activate the parasympathetic system to elicit physiological changes comparable to VR. Additionally, Rodríguez et al. (2024) found that even a single mindfulness session could significantly raise skin temperature (*p* < 0.05), suggesting that only deep and focused mind–body practices can effectively suppress sympathetic activity. In the standalone imagery dialogue group, participants received no external visual stimulation and therefore had to generate and maintain the natural scene largely through internal imagery. Compared with the VR-assisted conditions, which provided external visual anchors for imagery dialogue, this may have required greater imagery-generation effort and weakened the physiological relaxation response. This interpretation is consistent with the non-significant skin-temperature change in the imagery dialogue group, whereas skin temperature increased significantly in the VR-related groups. In summary, the lack of significance in the imagery dialogue group likely stemmed from insufficient stimulus intensity, limited immersion, and individual differences in imaginative ability. Conversely, the imagery dialogue-based virtual nature intervention integrated external virtual natural environments with internal guided imagery. It not only provided realistic natural landscapes but also offered emotional security, more effectively stimulating parasympathetic activity and leading to significant increases in skin temperature and relaxation. These mechanisms may explain why the VR-related conditions showed clearer skin-temperature changes than imagery dialogue alone in the present sample.

### 4.3 Comparison of intervention approaches and mechanisms

The core innovation of this study lies in the coordinated integration of virtual nature scenes and imagery dialogue within a rigorous mixed experimental design. The combined intervention showed the largest descriptive reduction in academic anxiety, although its statistical superiority over either single-component intervention was not established. This descriptive pattern suggests that multisensory relaxation environments and cognitive imagery techniques may make complementary contributions to anxiety regulation, although a synergistic effect was not directly established. Both the imagery dialogue group and the virtual nature group exhibited significant decreases in academic anxiety from pre-test to post-test. However, the present simple-effects analyses did not directly compare the magnitude of these decreases between the two groups. The observed pattern provides a basis for further examining whether integrating internal psychological adjustment with environmental relaxation offers additional benefits beyond either component alone.

The larger descriptive reduction observed in the combined intervention may be related to its coordinated engagement of cognitive-emotional and physiological-environmental components of anxiety. Mechanistically, virtual nature offers a safe and immersive setting that reduces initial physiological arousal and tension, laying the groundwork for psychological intervention. Immersive VR enhances presence and focus, helping participants “let go” of real-world distractions and concentrate on the relaxation experience (Jo et al., 2024). Studies have shown that VR enhances attention and immersion during meditation or relaxation, amplifying the intervention’s effects (Bosman et al., 2024). This may be particularly relevant for individuals who experience difficulty generating imagery or maintaining attention, because the visual and auditory cues provided by VR can support sustained engagement with the intervention.

Simultaneously, imagery dialogue is intended to engage the cognitive-emotional representations associated with anxiety by guiding individuals to interact with their anxiety sources and reconstruct negative academic scenarios into more positive imagery. This reimagining process helps reduce fear and helplessness associated with academic pressure (Maier et al., 2020). When such imagery is conducted within a peaceful VR nature setting, its effects may be further enhanced. The vivid contextual and sensory cues provided by VR may enhance the experiential quality of imagery dialogue and facilitate engagement with imagery-based emotional processing (Seinfeld and Montesano, 2025). As some scholars have noted, VR significantly expands the depth and breadth of psychological interventions by eliciting vivid emotional responses and constructing immersive contexts. In the combined intervention, the calming virtual natural scenes may have provided a supportive context for participants to engage with imagery dialogue. Within this context, imagery dialogue provided a structured means of reframing anxiety-related academic experiences. This coordinated format may integrate environmental relaxation with cognitive-emotional processing, although these underlying processes were not directly measured in the present study.

In summary, imagery dialogue and virtual nature emphasize different aspects of intervention: the former focuses on cognitive-emotional coping, whereas the latter emphasizes physiological and environmental regulation. Their integration may therefore provide a complementary and multimodal intervention framework that coordinates physiological-environmental regulation with cognitive-emotional processing.

## 5 Limitations

Despite the positive outcomes of this study, several limitations should be noted. First, the sample size was relatively small, and participants were primarily university students from a single region, limiting the generalizability of the findings across different geographic and cultural contexts. Future studies should aim to include larger and more diverse samples, such as students from different universities, academic disciplines, and grade levels. It may also be beneficial to extend the research to other populations, such as high school or graduate students, to examine the applicability of the findings across broader groups. Second, the intervention period was relatively short, focusing only on immediate pre- and post-intervention effects without tracking long-term outcomes. Given the recurring nature of academic anxiety, future research should incorporate follow-up assessments several weeks or months after the intervention to evaluate the maintenance and long-term benefits of the treatment. Furthermore, in terms of the control group setup, the no-intervention group used in this study could only partially control for maturation effects, but could not effectively control for the placebo effect, demand characteristics, or participants’ subjective expectations. Future research should introduce positive control conditions (such as passively watching natural landscape videos on a 2D screen) to more accurately isolate and confirm the specific active mechanisms of VR and imagery dialogue. Finally, there is room for discussion regarding the dynamic tracking frequency of physiological data. For the sake of standardizing the experimental procedure, this study collected physiological indicators at the beginning and end of the three-week intervention. The significant improvements in heart rate and skin temperature observed in this study reflect both the cumulative physiological adaptation mechanisms formed by individuals after multiple training sessions and the immediate relaxation response during the anxiety-induced and recovery tasks in the final assessment. Future research that incorporates continuous multi-point physiological monitoring during the intervention process will help to more accurately elucidate the temporal dynamic characteristics of this physiological remodeling. With advancements in technology and the growing accessibility of VR devices, future directions could also include the development of self-help VR-based relaxation and imagery training applications for students, enabling remote and daily use of the intervention. These directions could further expand the reach and impact of the current methodology and contribute to the development of innovative mental health interventions for college students.

## 6 Conclusion

This study showed that imagery dialogue, virtual nature exposure, and their integrated application were associated with reductions in academic anxiety among college students, with the combined condition showing the largest descriptive decrease. By coordinating the immersive, multisensory experience of VR with guided imagery dialogue, the combined protocol provided a structured context in which external sensory cues were aligned with imagery-based cognitive-emotional processing. The intervention groups showed significant reductions in academic anxiety, together with significant time × group interactions in heart rate and skin temperature. From a design perspective, integrating VR with imagery dialogue provides external visual and auditory cues that may strengthen situational vividness and reduce reliance on internally generated imagery during traditional imagery-based practice. This is consistent with a proposed theoretical mechanism in which multisensory stimulation and contextual engagement may contribute to anxiety reduction. The findings are consistent with recent evidence that VR-based mindfulness and virtual nature interventions may reduce anxiety and negative mood among university students (Ma et al., 2025a,b). Furthermore, the study has practical implications for mental health interventions in higher education settings. VR-based interventions may provide scalable and immersive experiences that complement existing counseling and mental health services in higher education settings (Zhang Q. et al., 2025). In the future, universities could evaluate the integration of this intervention paradigm into existing student mental health services. Immersive virtual natural scenes combined with guided imagery dialogue may offer students an accessible means of engaging with and regulating academic stress and anxiety, although its longer-term effectiveness should be established in further studies.

## Data Availability

The original contributions presented in the study are included in the article/supplementary material; further inquiries can be directed to the corresponding author.
